# Fabrication of Nitrogen‐Doped Mesoporous Carbon With Tunable Pore Size via Self‐Assembly of Poly(4‐Vinylpyridine)‐*Block*‐Poly(2,2,2‐Trifluoroethyl Methacrylate)

**DOI:** 10.1002/marc.202500529

**Published:** 2025-10-08

**Authors:** Youngwon Kong, Yuta Miyamori, Haruna Sasaki, Ryohei Kikuchi, Kan Hatakeyama‐Sato, Teruaki Hayakawa, Yuta Nabae

**Affiliations:** ^1^ Department of Materials Science and Engineering School of Materials and Chemical Technology Institute of Science Tokyo Meguro‐ku Tokyo Japan; ^2^ Materials Analysis Division Core Facility Center Research Infrastructure Management Center Institute of Science Tokyo Meguro‐ku Tokyo Japan; ^3^ Department of Technology Management for Innovation The University of Tokyo Bunkyo‐ku Tokyo Japan

**Keywords:** block copolymers, nitrogen‐doped mesoporous carbons, self‐assembly, soft‐template methods, tunable pore sizes

## Abstract

Precise control of pore structures of nitrogen‐doped mesoporous carbon (NMC) is still challenging. In this study, we address this issue by developing a soft‐template approach based on the molecular design of block copolymers, enabling systematic tuning of nanostructures. Specifically, we synthesized poly(4‐vinylpyridine)‐*block*‐poly(2,2,2‐trifluoroethyl methacrylate) (P4VP‐*b*‐PTFEMA) via reversible addition–fragmentation chain‐transfer (RAFT) polymerization and employed it as a soft template for fabricating NMCs. The P4VP block selectively interacts with phenol‐formaldehyde resol, enabling retention of microphase‐separated morphology during carbonization, while the fluorine‐containing PTFEMA block enhances phase separation through strong segmental repulsion. Ordered morphologies, including cylindrical structures, are formed upon blending with resol. These morphologies are preserved during thermal treatment at 900°C, leading to the formation of NMCs with well‐defined porous structures. The resulting NMCs exhibit tunable pore diameters ranging from 5.5 to 21.3 nm, controlled by the degree of polymerization of the PTFEMA block. These results highlight the potential of block copolymer design for achieving predictable mesopore architectures, offering a scalable platform for the development of functional porous materials.

## Graphical abstract

Nitrogen‐doped mesoporous carbons with tunable pore sizes were fabricated via self‐assembly of poly(4‐vinylpyridine)‐*block*‐poly(2,2,2‐trifluoroethyl methacrylate) and resol. Systematic variation of the block copolymer composition enabled precise control of the resulting cylindrical mesostructures, yielding pore diameters ranging from 5.5 to 21.3 nm. This molecular design strategy offers a versatile platform for tailored porous materials.

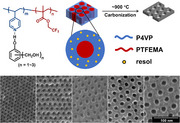

## 1 Introduction

Nitrogen‐doped mesoporous carbon (NMC) has attracted significant attention due to its excellent electrical conductivity and chemical stability, making it a promising material for various applications such as energy storage [1], supercapacitors [2, 3, 4], and electrocatalysis [5, 6, 7, 8]. One common method for fabricating NMC is the hard‐template method, which uses mesoporous silica as a structural template [7, 8, 9, 10]. In this process, a polymer precursor is infiltrated into the silica template and then carbonized. While this yields well‐defined carbon structures, it requires strong acids or bases such as hydrofluoric acid or sodium hydroxide to remove the silica, making the method complex and less efficient [11, 12].

To address these challenges, the soft‐template method has been developed. This approach utilizes the self‐assembly of block copolymers, which undergo microphase separation to form periodic structures ranging from 5 to 50 nm [13, 14, 15]. By introducing a crosslinker such as phenol‐formaldehyde resol, the microphase‐separated morphology can be preserved, enabling the fabrication of mesoporous carbon with well‐defined pores [16, 17]. Previous studies have reported the fabrication of mesoporous carbon materials using a variety of block copolymers, including poly(styrene)‐*block*‐poly(4‐vinylpyridine) (PS‐*b*‐P4VP) [18, 19, 20, 21], poly(ethylene oxide)‐*block*‐poly(ethyl acrylate)‐*block*‐PS (PEO‐*b*‐PEA‐*b*‐PS) [22], PEO‐*block*‐poly(caprolactone) [4, 23, 24], PS‐*block*‐PEO [25], and poly(isoprene)‐*block*‐PS‐*block*‐PEO [26], as well as commercially available copolymers such as Pluronic F127 [12, 27, 28, 29, 30, 31, 32, 33, 34, 35, 36, 37, 38, 39, 40, 41, 42, 43, 44, 45, 46, 47, 48, 49, 50, 51, 52, 53, 54, 55] and P123 [11]. In these studies, pore size was tuned mainly by adjusting processing parameters such as the polymer‐to‐precursor ratio [4, 28, 33, 40, 45, 46] or by varying the composition of block copolymers [56].

Among candidate soft templates, poly(4‐vinylpyridine)‐*block*‐poly(2,2,2‐trifluoroethyl methacrylate) (P4VP‐*b*‐PTFEMA) offers several distinct advantages. The P4VP block, which contains a nitrogen atom in the para position of the vinyl group, exhibits greater dipole polarization and a larger *χ* parameter compared to 2‐vinylpyridine, where the nitrogen atom is in the ortho position [57, 58]. This increased dipolar interaction enhances the block copolymer's compatibility with various additives, such as resorcinol [18] and phenol‐formaldehyde resol [56, 59, 60], primarily through hydrogen bonding between the pyridine units and hydroxyl groups [61, 62]. These interactions enable precise control over morphology, making P4VP‐*b*‐PTFEMA a versatile template for creating ordered structures in porous carbon materials. Additionally, the PTFEMA block, with its fluorine‐containing moiety, induces strong repulsion with other components, further promoting phase separation and enhancing the material's structural integrity during carbonization [63, 64, 65, 66]. This combination of functional groups provides a unique opportunity for designing nitrogen‐doped porous carbon materials with tunable properties at the molecular level.

**SCHEME 1:**
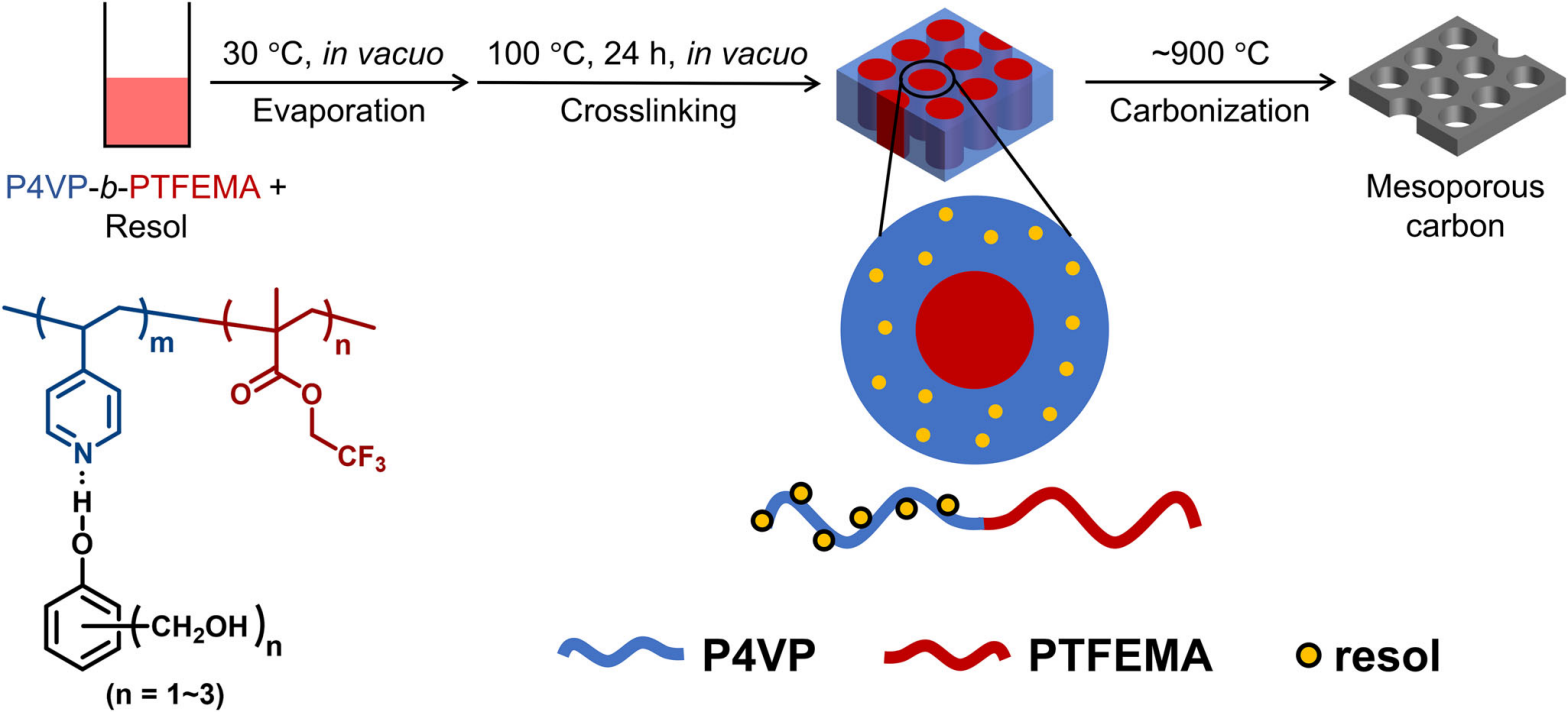
Schematic illustration of the preparation of P4VP‐*b*‐PTFEMA/resol blend samples and the fabrication of NMCs via thermal treatment.

Building on this molecular design, this study explores the fabrication of NMCs using a series of P4VP‐*b*‐PTFEMA block copolymers with varying degrees of polymerization. The block copolymers were synthesized via reversible addition–fragmentation chain‐transfer (RAFT) polymerization, and NMCs were prepared by blending them with resol. The solvent was first slowly removed at 30°C, and the resol was then crosslinked at around 100°C for 24 h to fix the structure. After crosslinking, the samples were carbonized under a two‐stage heating ramp, first at 1°C min^−1^ up to 600°C to prevent structural collapse during the early stage of carbonization, and then at 10°C min^−1^ up to 900°C to ensure efficient conversion to carbon while preserving nitrogen functionalities and maintaining a well‐ordered mesoporous structure [67], as illustrated in Scheme 1. We investigated how systematic changes in the block copolymer structure affect the resulting mesoporous morphology, particularly pore size, after thermal carbonization. In addition to conventional characterization techniques such as small‐angle X‐ray scattering (SAXS), scanning electron microscopy (SEM), transmission electron microscopy (TEM), and N_2_ adsorption, we implemented AI‐assisted image segmentation using the Segment Anything Model (SAM) to support structural analysis [68]. This integration of classic and modern approaches enabled a comprehensive understanding of how block copolymer design governs mesopore architecture. Through this combined approach, we reveal how tuning molecular parameters leads to predictable structural outcomes in soft‐template‐based NMCs—an insight that provides a blueprint for designing functional porous materials with customized architectures for next‐generation applications.

## 2 Results and Discussion

### 2.1 Preparation of P4VP‐*b*‐PTFEMA/Resol Blend Samples

P4VP‐*b*‐PTFEMA block copolymers were synthesized via RAFT polymerization according to our previously reported method [59]. To investigate the relationship between polymer composition and pore structure, six block copolymers with varying degrees of polymerization (DP) were prepared. The volume fraction of the P4VP block was designed to be approximately 0.6 to promote cylindrical morphology upon resol addition. The detailed polymerization conditions are summarized in Table S1. Successful synthesis was confirmed by ^1^H NMR spectroscopy and SEC, as shown in Figures S1 and S2, respectively. The compositions of the six block copolymers are summarized in Table 1.

**TABLE 1:** Compositions of synthesized P4VP‐*b*‐PTFEMA block copolymers.

Entry	*M*_n P4VP_ [kg mol^−1^]^a^	*M*_n PTFEMA_ [kg mol^−1^]^a^	*M*_n_ [kg mol^−1^]^a^	*Ð* ^b^	*φ*_P4VP_ ^a^
P4VP_43_‐*b*‐PTFEMA_20_	4.6	3.3	7.9	1.08	0.64
P4VP_95_‐*b*‐PTFEMA_64_	10.0	10.8	20.7	1.13	0.54
P4VP_135_‐*b*‐PTFEMA_94_	14.1	15.8	30.0	1.15	0.53
P4VP_213_‐*b*‐PTFEMA_121_	22.4	20.3	42.7	1.12	0.63
P4VP_317_‐*b*‐PTFEMA_145_	33.4	24.4	57.7	1.10	0.64
P4VP_477_‐*b*‐PTFEMA_218_	50.1	36.7	86.8	1.19	0.64

^a^ Determined by ^1^H NMR spectra in CDCl_3_;.

^b^ Determined by SEC with Shodex GPC LF‐804 column and 50 mm LiBr in DMF with relative to PS standards.

**FIGURE 1:**
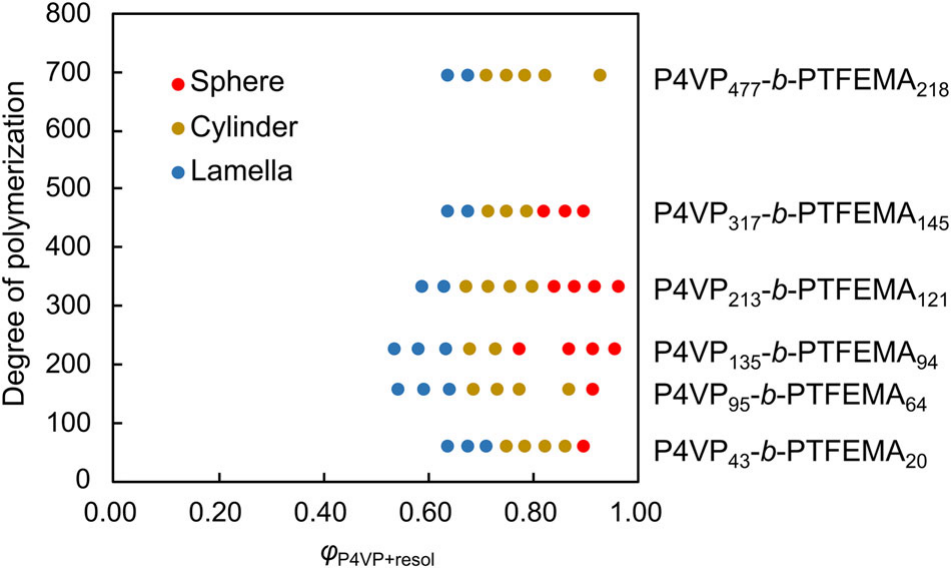
Compositional map of blend samples showing various morphology with resol addition. The x‐axis represents the combined volume fraction of P4VP and resol, and the y‐axis indicates the DP of the block copolymer. Each point corresponds to a specific blend sample, and the observed morphology was classified based on SAXS results (see Figure S3).

To explore the effect of resol content on the microphase‐separated structures, blend samples were prepared by varying the weight ratio of resol to block copolymer from 10 to 90 wt.%. The resulting microphase‐separated structures were analyzed by SAXS analysis. As shown in Figure 1, the microphase‐separated structures of all blend samples were summarized in a compositional map, where the x‐axis represents the volume fraction of P4VP and resol, and the y‐axis corresponds to the DP of the block copolymer. The SAXS profiles used to construct this map are provided in Figure S3. All the block copolymers without resol exhibited lamellar structures, as expected from their volume fractions and molecular characteristics. Upon gradual addition of resol, the morphology transitioned from lamellae to cylinders, and eventually to spheres at higher resol contents. This trend indicates that resol acts as a swelling agent for the P4VP domain, effectively tuning the volume fraction and inducing transitions in the microphase‐separated structures. As the resol content increased, the primary SAXS peak became progressively broader, and for samples containing more than 70 wt.% of resol, broad peaks overlapped with higher‐order reflections (Figure S3). This suggests that when a large amount of resol is added, a portion of the resol may no longer be fully miscible with the P4VP domain, leading to disruption of the ordered microphase‐separated structure.

**FIGURE 2:**
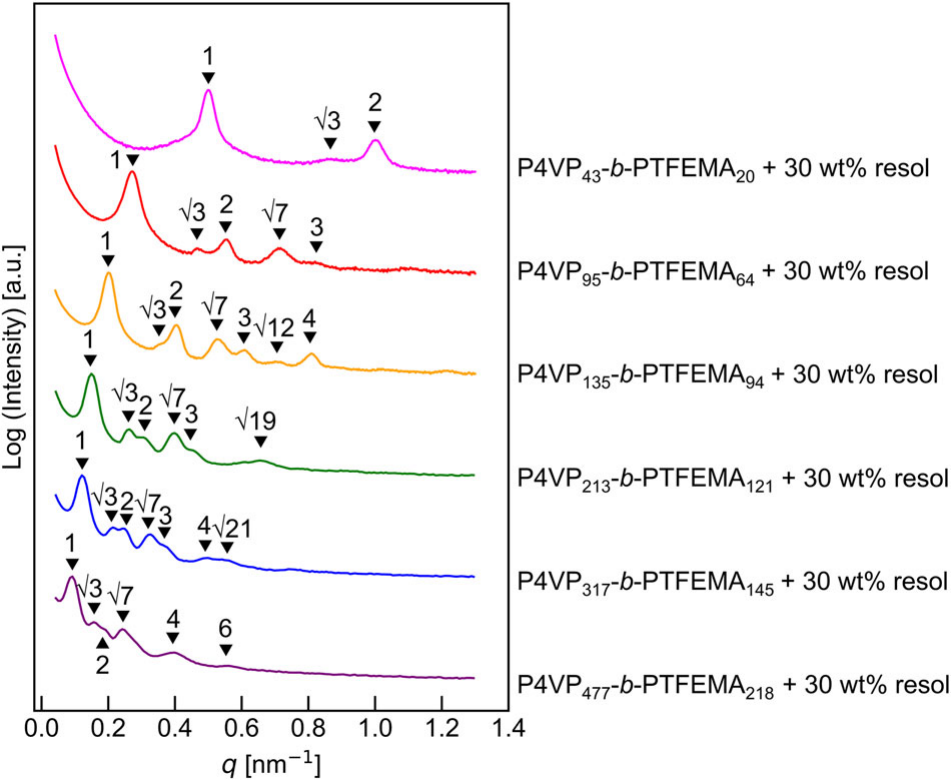
SAXS profiles of blend samples containing 30 wt.% resol for each of the six P4VP‐*b*‐PTFEMA block copolymers. All samples exhibit characteristic peaks corresponding to cylindrical morphology.

**FIGURE 3:**
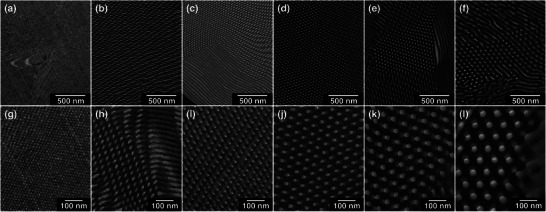
TEM images of blend samples of P4VP‐*b*‐PTFEMA block copolymers with 30 wt.% resol. Each column corresponds to a different block copolymer: (a), (g)—P4VP_43_‐*b*‐PTFEMA_20_, (b), (h)—P4VP_95_‐*b*‐PTFEMA_64_, (c), (i)—P4VP_135_‐*b*‐PTFEMA_94_, (d), (j)—P4VP_213_‐*b*‐PTFEMA_121_, (e), (k)—P4VP_317_‐*b*‐PTFEMA_145_, (f), (l)—P4VP_477_‐*b*‐PTFEMA_218_.

Based on the SAXS analysis as shown in Figure S3, we selected the samples that clearly exhibited cylindrical structures as the targets for carbonization. Figure 2 shows the SAXS profiles of the selected blend samples containing 30 wt.% resol for each of the six block copolymers, all of which exhibited well‐defined cylindrical morphologies. In addition, TEM images of the same blend samples are presented in Figure 3, where the hexagonally packed cylindrical structures are clearly observed for each sample. These images also illustrate that the *d*‐spacing increases with the molecular weight of the block copolymer, consistent with the trends observed in the SAXS data.

### 2.2 Structural Properties of NMCs

The blend samples containing 30 wt.% resol were subjected to thermal treatment to obtain cylindrical NMCs. Carbonization was conducted under a N_2_ atmosphere by heating the samples to 600°C at a rate of 1°C min^−1^, followed by further heating to 900°C at 10°C min^−1^. For clarity, the resulting nitrogen‐doped mesoporous carbon samples are labeled as NMC‐20, NMC‐64, NMC‐94, NMC‐121, NMC‐145, and NMC‐218, where the number indicates the DP of the PTFEMA block in the original block copolymer.

Elemental analysis revealed that 3.0% of nitrogen remained after carbonization, while no fluorine was detected (Table 2). This indicates that the PTFEMA block was fully decomposed during the heat treatment, and a portion of the P4VP block, which was miscible with resol, remained in the carbonized framework. The retained nitrogen atoms are considered to originate from this partially carbonized P4VP, resulting in successful nitrogen doping of the carbon structure.

**TABLE 2:** Elemental composition of the NMC‐64 before and after carbonization.

Entry	C [%]	H [%]	N [%]	F [%]
Before carbonization	65	6.1	6.8	8.8
After carbonization	92	0.9	3.0	N/D

**FIGURE 4:**
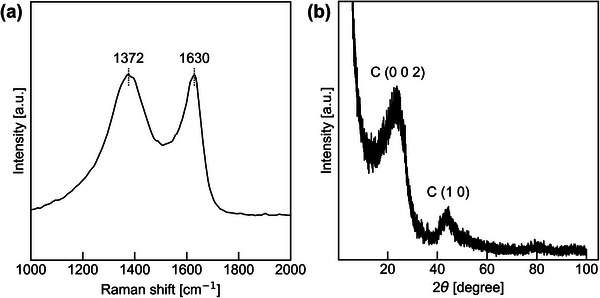
(a) Raman spectrum and (b) XRD pattern of the NMC‐64.

The structural characteristics of the obtained NMCs were further examined by Raman and XRD analyses, as shown in Figure 4a,b, respectively. The Raman spectrum exhibited two broad peaks centered at 1372 cm^−1^ (D band) and 1630 cm^−1^ (G band), with an intensity ratio (*I*_D_/*I*_G_) of approximately 1.0. This suggests the presence of a moderately disordered carbon structure, where graphitic and defective domains coexist. In the XRD pattern, a broad (002) diffraction peak was observed around 2*θ*  =  23°, along with a weak (100) peak near 44°. These features are characteristic of turbostratic carbon, where graphene‐like layers are stacked with random orientations and spacings, rather than forming an orderly layered structure like in crystalline graphite.

### 2.3 Pore Size Control by Tuning Composition of Block Copolymer

**FIGURE 5:**
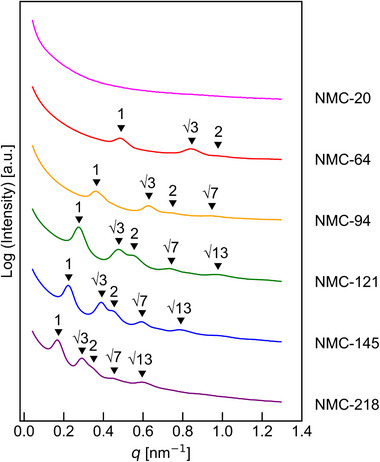
SAXS profiles of NMCs derived from blend samples with 30 wt.% resol.

To investigate the structural integrity and pore characteristics of NMCs, SAXS, N_2_ adsorption, and SEM analyses were conducted. The SAXS profiles of the carbonized samples are shown in Figure 5. Among the six block copolymers containing 30 wt.% resol tested, NMC‐20 exhibited no discernible structure after carbonization, suggesting a complete collapse of the cylindrical morphology during pyrolysis. In contrast, the other five samples successfully retained well‐defined cylindrical structures. Notably, these successful cases correspond to block copolymers with a P4VP block DP of 95 or higher, indicating that the critical threshold for preserving the mesostructure upon carbonization lies between P4VP block DPs of 43 and 95.

**FIGURE 6:**
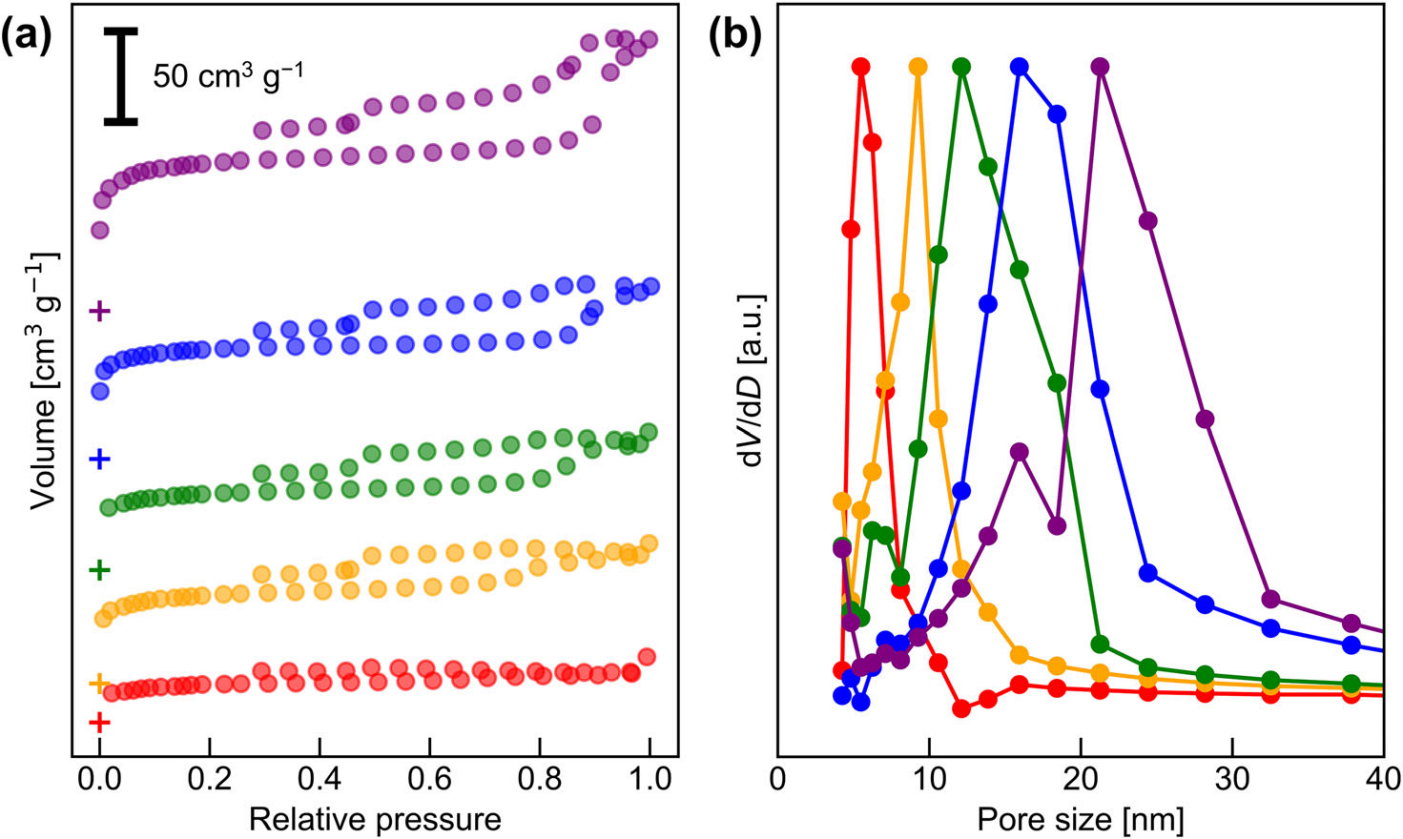
(a) N_2_ adsorption isotherms (with + symbols marking the (0, 0) points) and (b) pore size distributions (calculated by the BJH method) of the five NMC samples prepared with 30 wt.% resol. The plots correspond to the following NMCs: NMC‐64 (red), NMC‐94 (orange), NMC‐121 (green), NMC‐145 (blue), and NMC‐218 (purple).

**FIGURE 7:**
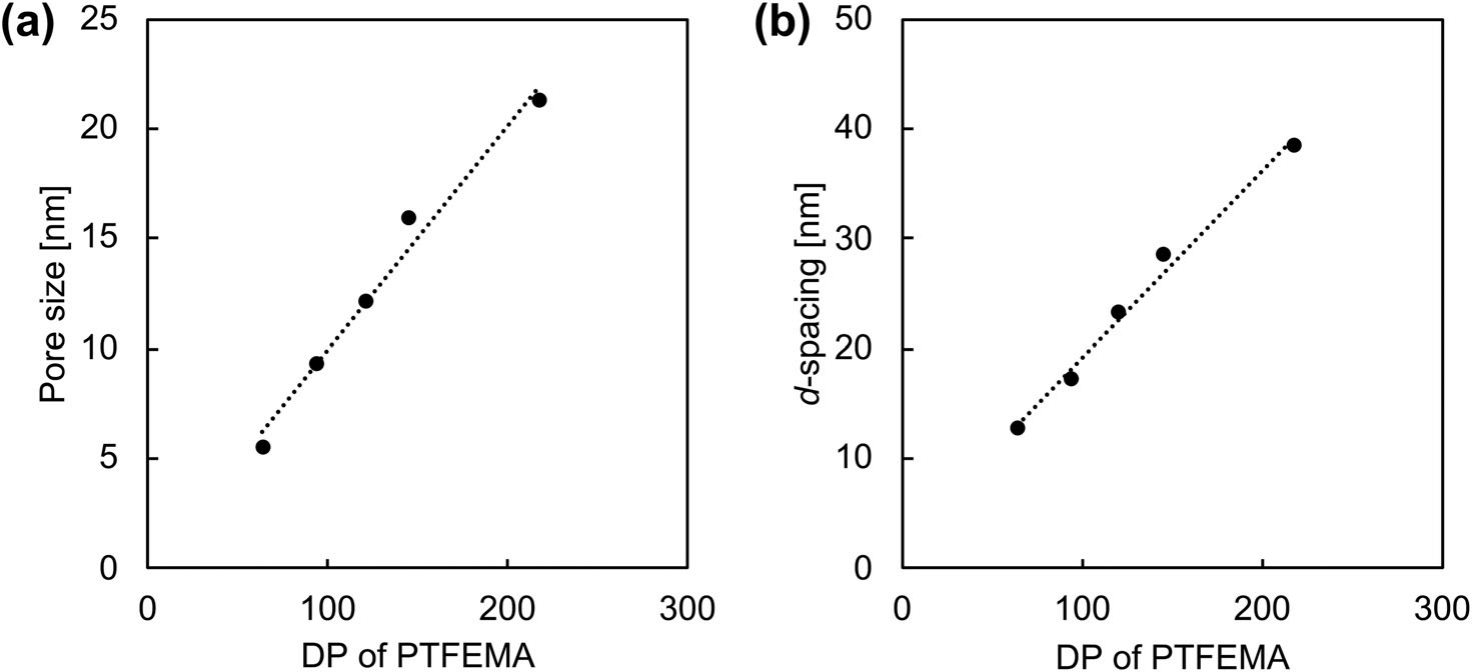
Correlation between DP of PTFEMA and (a) pore size derived from BJH method and (b) *d*‐spacing derived from SAXS measurements.

N_2_ adsorption measurements were performed on the same five carbonized samples. As shown in Figure 6, all samples exhibited type‐IV isotherms, confirming the formation of mesopores. The average pore diameters (*D*_pore_) estimated by the BJH method were as follows: NMC‐64 – 5.5 nm, NMC‐94 – 9.3 nm, NMC‐121 – 12.1 nm, NMC‐145 – 15.9 nm, NMC‐218 – 21.3 nm. These results indicate that the DP of the PTFEMA block strongly influences the final pore size of the NMCs. Since PTFEMA serves as the sacrificial domain that decomposes during carbonization, a higher DP corresponds to a larger decomposable volume, which directly contributes to the formation of larger mesopores. As shown in Figure 7, both the pore size determined from the BJH method (a) and the *d*‐spacing derived from SAXS measurements (b) exhibit clear correlations with the DP of PTFEMA. The agreement between these two independent measurements provides further confidence in the reliability of the pore size analysis and confirms that the DP of PTFEMA is a key parameter for controlling the mesoporous structure.

In addition to the pore size, the mesopore volume (*V*_meso_) also increased systematically with increasing *D*_pore_, as summarized in Table 3. This trend suggests that not only the size but also the abundance of mesopores increases with the molecular weight of the PTFEMA block, supporting the conclusion that both pore size and porosity can be effectively tuned through block copolymer design. Notably, as shown in Figure S4 and summarized in Table S2, the pore size range achieved in this study exceeds that of previously reported well‐ordered cylindrical NMCs. This broad tunability, achieved solely by adjusting the block copolymer composition, allows precise control over the mesoporous architecture to meet the specific requirements of various applications. For example, smaller pores may be advantageous for selective ion transport in electrochemical devices, whereas larger pores may facilitate mass transfer in catalytic systems. This flexibility highlights the potential of the present design strategy for developing application‐specific porous materials in fields such as energy storage, electrocatalysis, and molecular separation. Thus, our findings not only demonstrate a versatile approach to structural control but also suggest a pathway toward the rational design of functional carbon materials for emerging technologies.

**TABLE 3:** Summary of N_2_ adsorption analysis for the five NMC samples prepared with 30 wt.% resol.

Entry	*D*_pore_ [nm]^a^	*S*_BET_ [m^2^ g^−1^]^b^	*S*_meso_ [m^2^ g^−1^]^a^	*V*_total_ [cm^3^ g^−1^]^c^	*V*_meso_ [cm^3^ g^−1^]^a^
NMC‐64	5.5	70	10	0.049	0.025
NMC‐94	9.3	188	27	0.117	0.051
NMC‐121	12.1	160	23	0.115	0.058
NMC‐145	15.9	234	20	0.147	0.060
NMC‐218	21.3	318	35	0.233	0.119

^a^ Determined using the BJH method;.

^b^ Determined using the BET method;.

^c^ Determined from the adsorption volume at *p*/*p*_0_ = 0.99.

**FIGURE 8:**
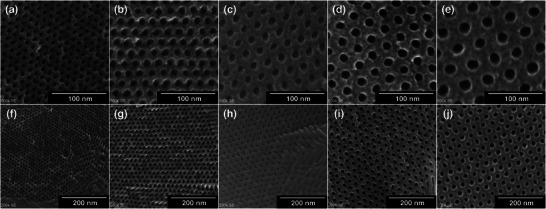
SEM images of NMCs obtained from five block copolymers. Each column corresponds to a different block copolymer: (a), (f)—NMC‐64 / (b), (g)—NMC‐94 / (c), (h)—NMC‐121 / (d), (i)—NMC‐145 / (e), (j)—NMC‐218.

The SEM images further supported these findings, as shown in Figure 8. All five NMCs exhibited porous structures consistent with cylindrical morphologies, and the apparent pore sizes observed were in good agreement with those estimated from N_2_ adsorption measurements. To estimate the pore and wall dimensions, the SEM images were analyzed using AI‐assisted image segmentation. Pore regions were identified, and the maximum inscribed circle within each region was used to estimate the pore diameter, while the center‐to‐center distance between neighboring pores provided an approximation of wall thickness (Figure S5). Although the SEM analysis may involve projection effects or slight tilt in the image acquisition, the trends observed were broadly consistent with the pore sizes and periodicities obtained by N_2_ adsorption and SAXS, respectively (Table S3 and Figure S6). These complementary results collectively support the preservation and tunability of cylindrical mesostructures in the NMCs.

## 3 Conclusion

In this study, NMCs were successfully fabricated using P4VP‐*b*‐PTFEMA as a tunable soft template and phenol‐formaldehyde resol as a crosslinkable precursor. The selective miscibility of the P4VP block with resol allowed the preservation of cylindrical microphase‐separated structures during carbonization, while the PTFEMA block decomposed to form mesopores.

By systematically varying the DP of the PTFEMA block, we achieved precise control over the mesopore size, which ranged from 5.5 to 21.3 nm. Notably, the mesostructure was maintained only when the P4VP block exceeded a critical chain length, underscoring the importance of molecular design in structural stability. The observed trends in pore size and morphology were consistently supported by SAXS, N_2_ adsorption, and SEM analyses, along with AI‐assisted image segmentation.

This study demonstrates a straightforward and scalable strategy for engineering NMCs with tunable pore dimensions and intrinsic nitrogen doping—without the use of additional nitrogen sources. The molecular‐level designability of P4VP‐*b*‐PTFEMA makes this soft‐template platform highly promising for the rational development of functional porous materials in applications such as energy storage and catalysis.

## 4 Experimental Section

### 4.1 Materials

2,2,2‐Trifluoroethyl methacrylate (TFEMA; Tokyo Chemical Industry; purity >98.0%) and 4‐vinylpyridine (4VP; Tokyo Chemical Industry; purity >95.0%) were purified using vacuum distillation over calcium hydride. 2,2’‐azobisisobutyronitrile (AIBN; Tokyo Chemical Industry; purity >98.0%) was purified by recrystallization from methanol before use. 2‐cyano‐2‐propyl benzodithioate (CPBD; Tokyo Chemical Industry; purity >95%), anhydrous tetrahydrofuran (THF; Kanto Chemical Co. Inc.; purity >99.5%), *N*,*N*‐dimethylformamide (DMF; Wako Chemicals Co.; purity >99.5%), *n*‐hexane, dichloromethane, phenol, formalin (37% formaldehyde solution in methanol), sodium hydroxide (NaOH), and hydrochloride (HCl) were used as purchased.

### 4.2 Characterization

Nuclear magnetic resonance (NMR) was conducted on a JEOL JNM‐ECS 400 (400 MHz) spectrometer using deuterated CHCl_3_ (CDCl_3_) at room temperature. The polydispersity (*Ð*) was evaluated in DMF with 50 mm LiBr at 40°C using a Viscotek GPCmax VE‐2001 system (Viscotek TDA 302 detector, Shodex LF 804 columns × 2) and calibrated with polystyrene (PS) standards. Small‐angle X‐ray scattering (SAXS) was performed on a Rigaku NanoPIX (40 kV/30 mA) using a sample‐to‐detector distance of 1.4 m and wavelength of 1.54 Å. The scattering vector (*q*) can be expressed as *q* = 4π sin(*θ*)/*λ*, where *θ* and *λ* are the scattering angle and wavelength, respectively. The porosity of the prepared samples was characterized by N_2_ adsorption using a BEL Japan Belsorp mini II and analyzed by the Barrett‐Joyner‐Halenda (BJH) method. NMCs were thermal treated at 350°C for 1.5 h under a vacuum to eliminate impurities like water before N_2_ adsorption measurement. Elemental analysis was carried out CHN analyzer (JM10 provided by J‐Science), and an ion chromatograph system (ICS1100 provided by Thermo Fisher Scientific) for quantifying fluorine. The field emission scanning electron microscope (SEM) images of the prepared samples were measured using a HITACHI S‐5500 microscope. Raman spectroscopy was performed using a JASCO RMP‐510 spectrometer. X‐ray diffraction (XRD) was performed using a Rigaku Ultima IV diffractometer with CuK*α* radiation. Transmission electron microscopy (TEM) was performed using an H‐7650 microscope (Hitachi High‐Tech) at 100 kV. The TEM samples were ultra‐sectioned using a Leica Microsystems EM UC7 at room temperature and collected onto microgrids. The grids and a small amount of iodine crystals were sealed in a glass vial and left at room temperature for 30 min to selectively stain the P4VP block.

Image‐based analysis of the pore diameter and wall thickness was conducted using the Segment Anything Model (SAM), a deep learning‐based image segmentation model developed by Meta AI [68]. SEM images were processed using a custom Python pipeline that integrates the SAM (ViT‐H model) with OpenCV‐based geometric analysis. Prior to segmentation, the top 10% of each SEM image was cropped to remove scale bars and labels. The SAM‐generated masks were used to extract individual pore regions, and only those within a specified size range (5–30 nm in diameter) were included in the analysis. The maximum inscribed circle within each segmented region was used to estimate the pore diameter. Wall thickness was calculated by identifying the nearest neighboring pore for each particle and subtracting the radii of the two from the center‐to‐center distance. Data that exceeded a defined area ratio threshold (outer/inner > 2.0) were excluded to eliminate irregular regions.

### 4.3 Preparation of P4VP‐*b*‐PTFEMA/Resol Blend Sample

A typical P4VP‐*b*‐PTFEMA sample was synthesized via RAFT polymerization. The detailed synthesis procedures for P4VP‐*b*‐PTFEMA and resol are provided in the Supporting Information. P4VP‐*b*‐PTFEMA and resol were dissolved in DMF at a concentration of 5 wt.% and placed in a sample tube. The solution was then evaporated under vacuum on a hot plate set to 30°C for approximately 4–5 days. After evaporation, the mixture was crosslinked by heating under vacuum at 100°C for 24 h.

### 4.4 Fabrication of NMC by Thermal Treatment

NMCs were fabricated by thermally treating blend samples of P4VP‐*b*‐PTFEMA and resol. The samples were heated under a N_2_ flow of 1.2 L min^−1^. The temperature was ramped at 1°C min^−1^ up to 600°C, then at 10°C min^−1^ to 900°C, followed by maintaining at 900°C for 1 h.

## Conflicts of Interest

The authors declare no conflicts of interest.

## Supporting information




**Supporting File**: marc70092‐sup‐0001‐SuppMat.docx.

## Data Availability

The data supporting this article has been included in the Supporting Information.
