# Supplementary material for: Fabrication of Nitrogen‐Doped Mesoporous Carbon With Tunable Pore Size via Self‐Assembly of Poly(4‐Vinylpyridine)‐Block‐Poly(2,2,2‐Trifluoroethyl Methacrylate)
Source: Macromol Rapid Commun. 2025 Oct 8;46(24):e00529. doi: 10.1002/marc.202500529 (PMC12713617; doi:10.1002/marc.202500529)
Supplement: Supplementary file 1 — Supporting File: marc70092‐sup‐0001‐SuppMat.docx. [file MARC-46-e00529-s001.docx]

Supporting Information

Fabrication of Nitrogen-Doped Mesoporous Carbon with Tunable Pore Size via Self-Assembly of Poly(4-Vinylpyridine)-*block*-Poly(2,2,2-Trifluoroethyl Methacrylate)

Youngwon Kong, Yuta Miyamori, Haruna Sasaki, Ryohei Kikuchi, Kan Hatakeyama-Sato,
Teruaki Hayakawa*, and Yuta Nabae*

**Synthesis of PTFEMA using reversible addition–fragmentation chain-transfer (RAFT) polymerization**

A typical PTFEMA was synthesized by mixing 2,2’-azobisisobutyronitrile (AIBN), 2-cyano-2-propyl benzodithioate (CPBD), and 2,2,2-trifluoroethyl methacrylate (TFEMA), and then degassing the mixture using five freeze-evacuate-thaw cycles and purging with argon gas. The mixture was then polymerized at 70 °C for 24 h, and the crude product was diluted with tetrahydrofuran (THF) and reprecipitated in *n*-hexane, followed by vacuum filtration. The resulting product was dried *in vacuo* at 40 °C overnight to yield PTFEMA as a pale pink powder.

**Synthesis of P4VP-*b*-PTFEMA using RAFT polymerization**

Typical P4VP-*b*-PTFEMA was synthesized by adding AIBN, PTFEMA, and 4-vinylpyridine (4VP) into THF. This solution was then degassed using five freeze-evacuate-thaw cycles and purged with argon gas. The mixture was subsequently polymerized at 70 °C for 24 h, and the crude product was diluted with dichloromethane and reprecipitated in *n*-hexane, followed by vacuum filtration. The resulting product was dried *in vacuo* at 40 °C overnight to yield P4VP-*b*-PTFEMA as a light pink powder. The synthesis conditions, including reaction time (2–24 h) and 4VP/PTFEMA molar ratio (1:300–800), were controlled to obtain P4VP-*b*-PTFEMA samples with various compositions (**Table S1**).

**Synthesis of phenol-formaldehyde resol**

A typical phenol-formaldehyde resol was synthesized by first melting 2.40 g (25.5 mmol) of phenol at 40 °C in a flask and then mixing it with 0.42 mL of a 20 wt% NaOH aqueous solution under a nitrogen atmosphere. After 10 min, 3.77 mL (37.8 mmol) of formalin (37 wt% formaldehyde solution) was added dropwise while maintaining the temperature below 40 °C. The mixture was then stirred at 70 °C for 1 h under a nitrogen atmosphere, resulting in a light orange mixture. The pH of the mixture cooled to room temperature was adjusted to approximately 7.0 using a 0.6 M hydrochloride (HCl) solution. Moreover, water was removed using vacuum evaporation below 50 °C. THF was then added to the mixture, and the white NaCl precipitate was removed by filtration. THF was removed by vacuum evaporation below 50 °C, and the product was dried under vacuum at 40 °C overnight, yielding 2.95 g of a light orange, high-viscosity solution.

Table S1. Polymerization conditions used for the synthesis of P4VP-*b*-PTFEMA block copolymers.

| Block copolymer | PTFEMA | | Time [h] | *M*_n_ [kg mol^−1^]^a)^ | *Ð*^c)^ | *φ*_P4VP_^a)^ |
| --- | --- | --- | --- | --- | --- | --- |
|  | *M*_n_ [kg mol^−1^]^a)^ | eq.^b)^ |  |  |  |  |
| P4VP_43_-*b*-PTFEMA_20_ | 3.3 | 300 | 2 | 7.9 | 1.08 | 0.64 |
| P4VP_95_-*b*-PTFEMA_64_ | 10.8 | 300 | 6 | 20.7 | 1.13 | 0.54 |
| P4VP_135_-*b*-PTFEMA_94_ | 15.8 | 450 | 6 | 30.0 | 1.15 | 0.53 |
| P4VP_213_-*b*-PTFEMA_121_ | 20.3 | 450 | 18 | 42.7 | 1.12 | 0.63 |
| P4VP_317_-*b*-PTFEMA_145_ | 24.4 | 600 | 18 | 57.7 | 1.10 | 0.64 |
| P4VP_477_-*b*-PTFEMA_218_ | 36.7 | 800 | 24 | 86.8 | 1.19 | 0.64 |

^a)^Determined by ^1^H NMR spectra in CDCl_3_; ^b)^Equivalence of 4VP (monomer) to PTFEMA (macro-chain transfer agent); ^c)^Determined by SEC with Shodex GPC LF-804 column and 50 mM LiBr in DMF with relative to PS standards.


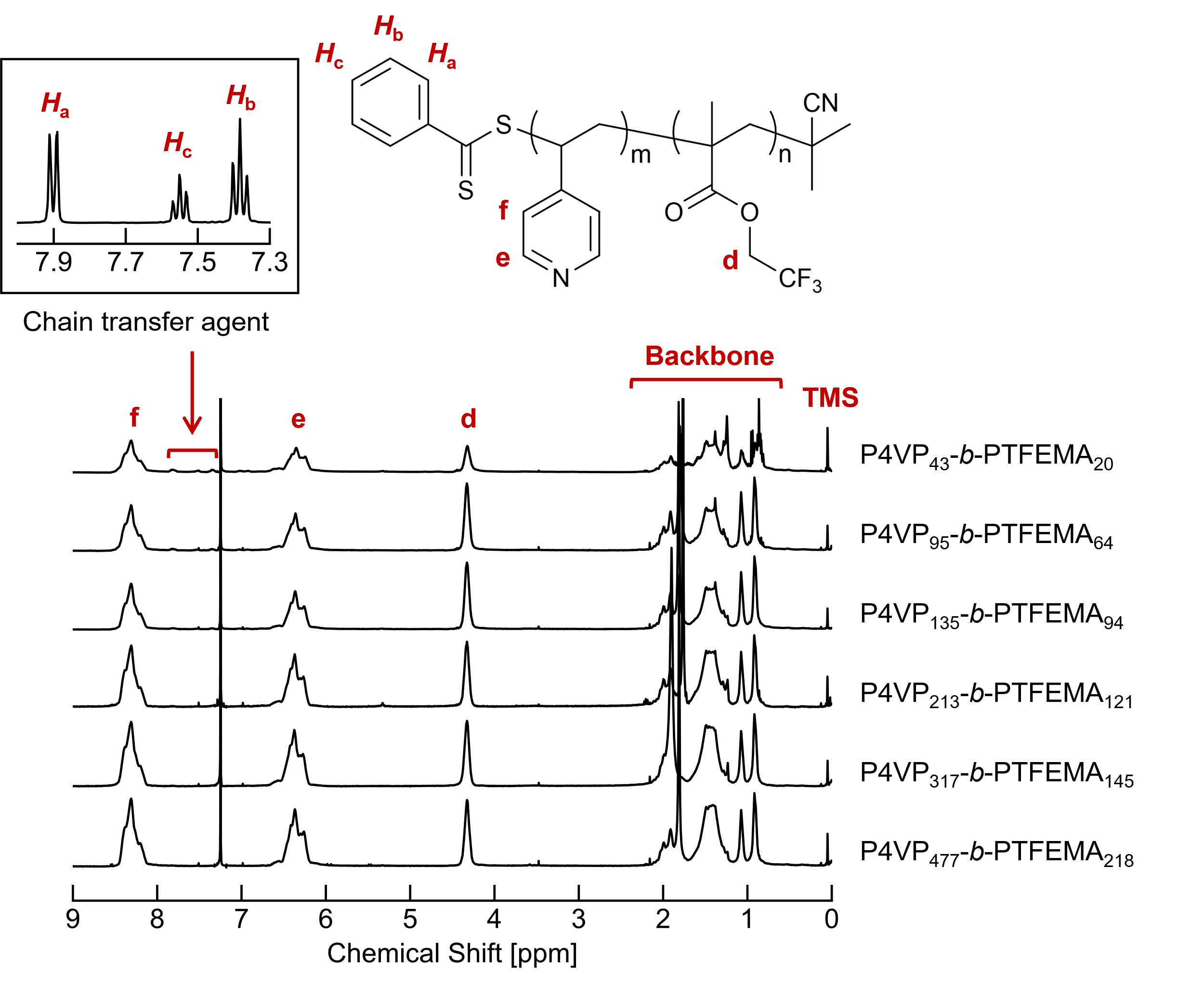


Figure S1. ^1^H NMR spectra of the synthesized P4VP-*b*-PTFEMA block copolymers.


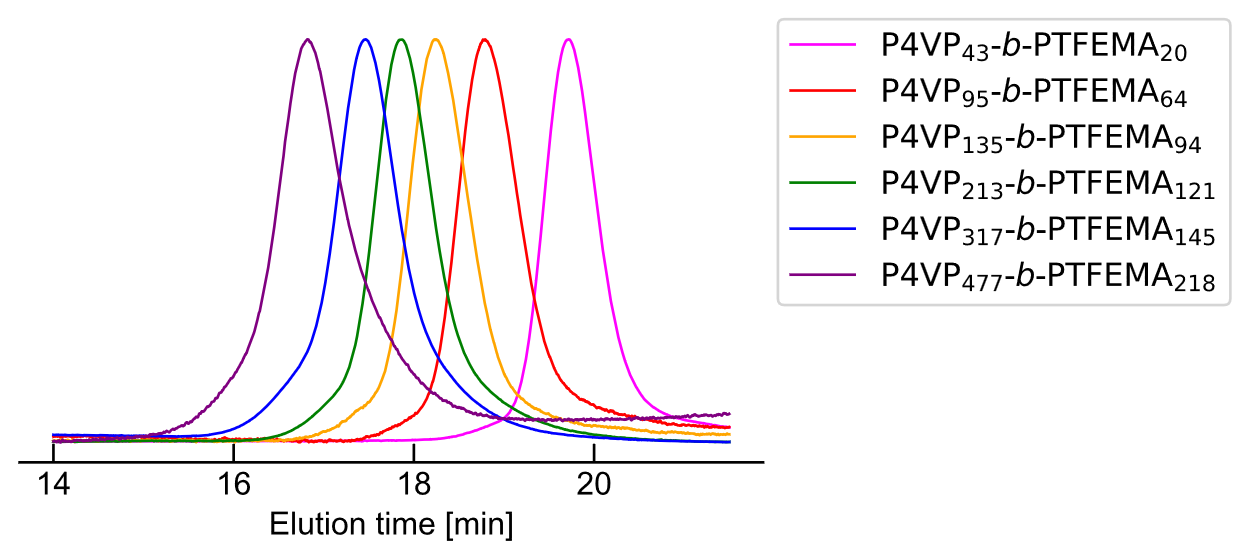


Figure S2. SEC chromatograms of P4VP-*b*-PTFEMA block copolymers.


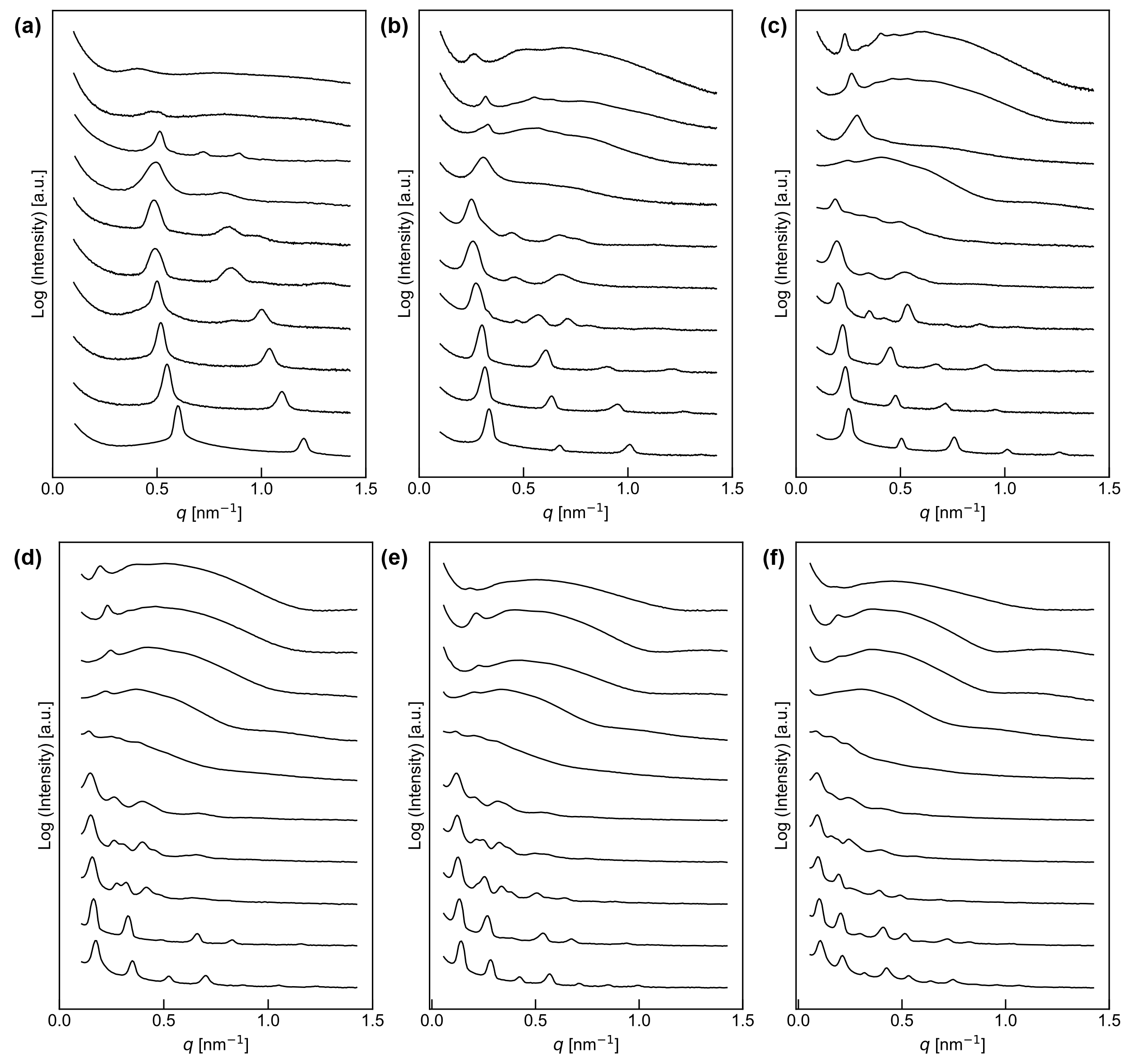


Figure S3. SAXS profiles of blend samples with increasing resol content (0–90 wt%) from bottom to top, for six different P4VP-*b*-PTFEMA block copolymers: (a) P4VP_43_-*b*-PTFEMA_20_, (b) P4VP_95_-*b*-PTFEMA_64_, (c) P4VP_135_-*b*-PTFEMA_94_, (d) P4VP_213_-*b*-PTFEMA_121_, (e) P4VP_317_-*b*-PTFEMA_145_, (f) P4VP_477_-*b*-PTFEMA_218_.


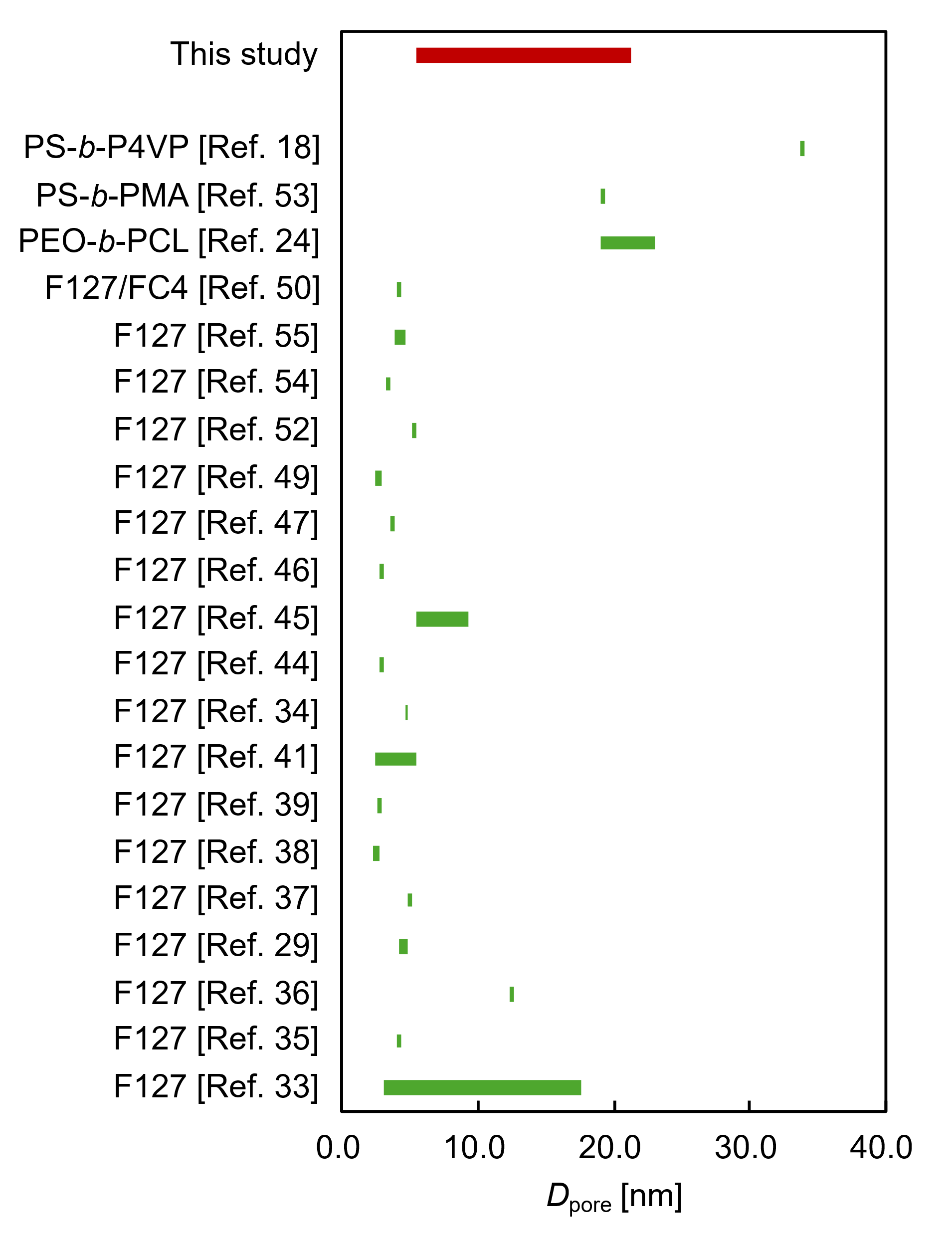


Figure S4. Comparison of pore size ranges in well-ordered cylindrical NMCs reported in previous studies and in this work.

Table S2. Summary of well-ordered cylindrical NMCs reported in the literature, including block copolymer templates, precursors, pore size ranges, nitrogen content, BET surface area

| Template | Precursor | *D*_pore_ | N content [%] | *S*_BET_ [m^2^ g^−1^] | Ref. |
| --- | --- | --- | --- | --- | --- |
| P4VP-*b*-PTFEMA | Resol | 5.5–21.3 | 3 | 70–318 | This study |
| PS-*b*-P4VP | Resorcinol/Formaldehyde | 33.7 | – | – | [18] |
| PS-*b*-PMA | Resol/Amic acid | 19.0 | – | – | [53] |
| PEO-*b*-PCL | N-Resol | 19.0–23.0 | 11.5–13 | 491–610 | [24] |
| F127/FC4 | Resorcinol/Formaldehyde/TMB | 4.0 | 5.5–25.7 | 307–484 | [50] |
| F127 | Resol/DADC | 3.1–17.6 | 13.1 | 494–586 | [33] |
| F127 | Resol/DCDA | 4.1 | 4.6 | 516 | [35] |
| F127 | Resol/Melamine | 12.3 | 11.6 | 320 | [36] |
| F127 | Resol/Amic acid | 4.2–4.8 | 2 | 770–1660 | [29] |
| F127 | Resol/Amic acid | 4.8 | 1.6 | 692 | [37] |
| F127 | Resol/Urea/PdCl_2_ | 2.2–2.8 | 0–12 | 480–538 | [38] |
| F127 | Aminophenol/HMTA | 2.6 | 4.3 | 1207 | [39] |
| F127 | Resol/Melamine Resin | 2.5–5.5 | 2.9 | 1166 | [41] |
| F127 | Resol/Dicyandiamide | 4.6 | 7.51 | 1344 | [34] |
| F127 | Resorcinol/Melamine/HMT | 2.7 | 2.36 | 631 | [44] |
| F127 | Resorcinol/Formaldehyde/Dicyandimide | 5.5–9.3 | 0.23–4.20 | 376–718 | [45] |
| F127 | UPF resin | 2.8 | 0.38–1.4 | 446–549 | [46] |
| F127 | UPF resin | 3.6 | 3.85 | 537 | [47] |
| F127 | Resol/Melamine | 2.5–2.9 | 18 | 386 | [49] |
| F127 | Resol/Dicyandiamide | 5.1 | 7 | 631 | [52] |
| F127 | 3-Aminophenol/HMT | 3.3 | 3.5–9.2 | 433 | [54] |
| F127 | Resol/TiCl_4_/Ni(NO_3_)_2_ | 3.8–4.6 | – | 319–430 | [55] |


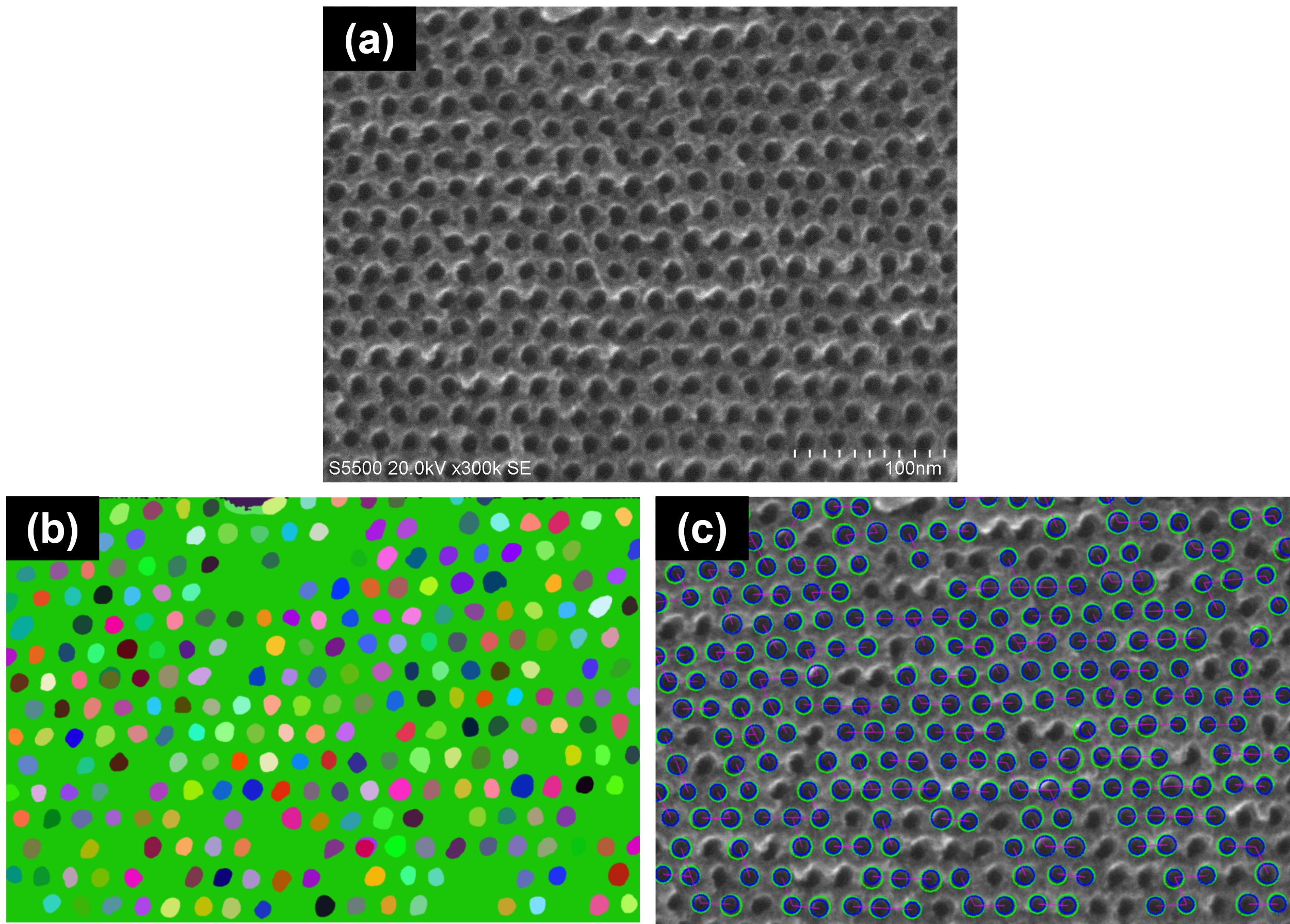


Figure S5. Representative images used for AI-assisted image segmentation of pore diameter and wall thickness. (a) Original SEM image of a NMC sample. (b) Corresponding Segment Anything Model (SAM)-generated mask highlighting pore regions. (c) Image overlay showing the maximum inscribed circles (blue), the circumscribed circles (green), and wall thickness estimations (pink lines) based on the center-to-center distances between adjacent pores.

Table S3. Summary of structural parameters of NMCs

| Entry | Block copolymer | *d*-spacing [nm]^a)^ | Pore diameter [nm]^b)^ | Wall thickness [nm]^b)^ |
| --- | --- | --- | --- | --- |
| NMC-64 | P4VP_95_-*b*-PTFEMA_64_ | 12.8 | 6.5 | 7.2 |
| NMC-94 | P4VP_135_-*b*-PTFEMA_94_ | 17.3 | 10.8 | 9.0 |
| NMC-121 | P4VP_213_-*b*-PTFEMA_121_ | 23.2 | 11.1 | 14.5 |
| NMC-145 | P4VP_317_-*b*-PTFEMA_145_ | 28.5 | 13.1 | 15.8 |
| NMC-218 | P4VP_725_-*b*-PTFEMA_197_ | 38.4 | 16.6 | 21.3 |

^a)^Determined by SAXS measurement; ^b)^Determined by image segmentation-estimated analysis of SEM images.


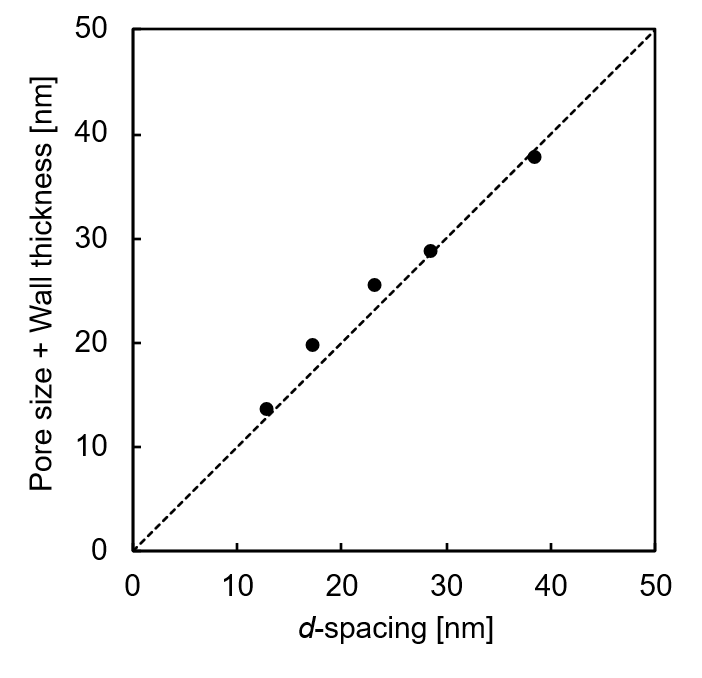


Figure S6. Correlation between SAXS-derived *d*-spacing and the sum of image segmentation-estimated pore diameter and wall thickness.
